# Neurogenic Bowel in Acute Rehabilitation Following Spinal Cord Injury: Impact of Laxatives and Opioids

**DOI:** 10.3390/jcm10081673

**Published:** 2021-04-14

**Authors:** Andrew M. Round, Min Cheol Joo, Carolyn M. Barakso, Nader Fallah, Vanessa K. Noonan, Andrei V. Krassioukov

**Affiliations:** 1 International Collaboration on Repair Discoveries (ICORD), Department of Medicine, University of British Columbia, Vancouver, BC V5Z 1M9, Canada; drewround@gmail.com (A.M.R.); cbarakso@alumni.ubc.ca (C.M.B.); vnoonan@praxisinstitute.org (V.K.N.); 2 Department of Medicine, University of British Columbia, Vancouver, BC V5Z 1M9, Canada; nfallah@praxisinstitute.org; 3 Department of Physical Medicine and Rehabilitation, University of Ottawa, Ottawa, ON K1H 8M2, Canada; 4 Department of Rehabilitation Medicine and Institute of Wonkwang Medical Science, Wonkwang University School of Medicine, Iksan 570-749, Korea; jmc77@daum.net; 5 Praxis Spinal Cord Institute, Vancouver, BC V5Z 1M9, Canada; 6 Division of Physical Medicine and Rehabilitation, Department of Medicine, University of British Columbia, Vancouver, BC V5Z 2G9, Canada; 7 GF Strong Rehabilitation Centre, Vancouver Coastal Health, Vancouver, BC V5Z 2G9, Canada

**Keywords:** bowel dysfunction, acute rehabilitation, spinal cord injury, laxatives, opioids, SCI bowel management

## Abstract

Objective: To explore the association between bowel dysfunction and use of laxatives and opioids in an acute rehabilitation setting following spinal cord injury (SCI). Methods: Data was collected regarding individuals with acute traumatic/non-traumatic SCI over a two-year period (2012–2013) during both the week of admission and discharge of their inpatient stay. Results: An increase in frequency of bowel movement (BM) (*p* = 0.003) and a decrease in frequency of fecal incontinence (FI) per week (*p* < 0.001) between admission and discharge was found across all participants. There was a reduction in the number of individuals using laxatives (*p* = 0.004) as well as the number of unique laxatives taken (*p* < 0.001) between admission and discharge in our cohort. The number of individuals using opioids and the average dose of opioids in morphine milligram equivalents (MME) from admission to discharge were significantly reduced (*p* = 0.001 and *p* = 0.02, respectively). There was a positive correlation between the number of laxatives and frequency of FI at discharge (r = 0.194, *p* = 0.014), suggesting that an increase in laxative use results in an increased frequency of FI. Finally, there was a significant negative correlation between average dose of opioids (MME) and frequency of BM at discharge, confirming the constipating effect of opioids (r = −0.20, *p* = 0.009).

## 1. Introduction

Spinal cord injury (SCI) is a devastating event, which affects multiple facets of an individual’s life with far-reaching implications and dangerous complications. Although paralysis is the most obvious and visible outcome of SCI, individuals have reported neurogenic bowel dysfunction (NBD) as being one of the greatest contributors to disability [1,2,3,4,5]. NBD occurs in up to 80% of individuals with SCI and has significant negative impacts on quality of life [6,7]. Bowel management is a key concern in this population as it frequently interferes with independence, causes embarrassment and social isolation, and alters relationships [8,9]. 

**Figure 1:**
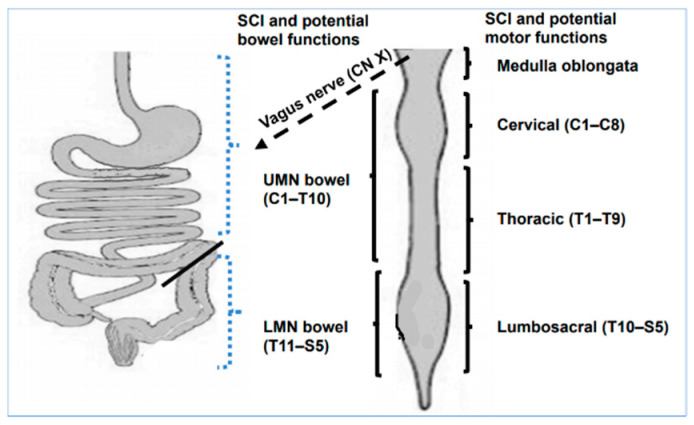
Schematic diagram of the gastrointestinal tract innervation and potential functional bowel and motor outcomes.

Bowel dysfunction following SCI is closely correlated with the level and severity of SCI, involving impaired abdominal and pelvic floor muscle control, impaired rectal sensation, and delayed colonic transit time (CTT) [10]. Two main patterns of bowel dysfunction in individuals with SCI who have recovered from spinal shock have been described in detail, known as upper motor neuron (UMN) and lower motor neuron (LMN) bowel syndromes [4] (Figure 1). UMN bowel syndrome, otherwise known as hyperreflexic bowel, is attributed to supraconal injury and has the predominant feature of constipation. Clinically, these individuals suffer from significant constipation and fecal retention with a reliance on rectal irritation to encourage stool propulsion via intact enteric/sacral reflexes. LMN bowel syndrome, otherwise known as areflexic bowel, is attributed to injury at the conus or cauda equina level and has the predominant features of fecal incontinence (FI) and decreased frequency of bowel movement (BM) due to constipation. Constipation is most often defined as fewer than two to three bowel movements per week [3,11]. These issues are explained by atonic external anal sphincter (EAS) and pelvic floor musculature secondary to disrupted alpha motor neurons in the sacral spinal segments. 

Innervation of GI tract: Parasympathetic innervation to the portion of the GI tract extending from the esophagus to the splenic flexure of the colon (solid line), which modulates peristalsis, is provided by the vagus nerve (CN X). Parasympathetic innervation to the descending colon and rectum is provided by the pelvic splanchnic nerves, which exit from the spinal cord at segments S2–S4. Sympathetic innervation to the upper GI tract is provided by the sympathetic preganglionic neurons (SPN) localized within the upper thoracic spinal cord segments (T1–T5); the small and large intestine are controlled by SPNs localized within the T6–L2 spinal segments. Somatic innervation and voluntary control of the external anal sphincter and pelvic floor musculature is originating from S2–S4 spinal cord segments.

Bowel functional outcomes and level of SCI: A SCI that damages segments above the sacral segments (above T10) produces a hyperreflexive or UMN bowel, in which defecation cannot be initiated by voluntary relaxation of the external anal sphincter, although there can be reflex-mediated colonic peristalsis. In contrast, a SCI that includes destruction of the lumbar/sacral spinal cord segments produces an areflexic or LMN bowel, in which there is no reflex-mediated colonic peristalsis. The anal sphincter of an LMN bowel is typically atonic and prone to leakage of stool.

Motor functional outcomes and SCI: Functional abilities of individuals with cervical and thoracic SCI ranges significantly. Meaningful functional categories include: independence in activities of daily living, wheelchair independence, bed mobility, voluntary weight shifting, and independent transfers. However, most of these individuals do not have abdominal, pelvic muscle or anal sphincter control. Finally, although individuals with lower thoracic/lumbar and sacral SCI (below T11 spinal segment) have full control of their upper extremities, core/abdominal musculature and may be able to stand or ambulate with assistive devices, individuals with complete spinal cord lesion do not have control to their pelvic floor muscles or anal sphincter. 

Abbreviations: C—cervical; T—thoracic; L—Lumbar; S—sacral; GI—gastrointestinal; SCI—spinal cord injury; SPNs—sympathetic preganglionic neurons; UMN—upper motor neuron; LMN—lower motor neuron.

Strict and detailed bowel management protocols delineated by UMN and LMN injuries have become recognized as essential in management of NBD in individuals with SCI [12]. The main goal of such regimens is to achieve a regular and efficient bowel evacuation within a reasonable and regular time frame [4,13]. The components of a successful bowel protocol commonly include regulation of diet (fiber intake), abdominal massage, digital rectal stimulation, manual evacuation, oral laxatives, transanal irrigation, rectal suppository, and other pharmacological agents (stool softeners, colonic stimulants, contact irritants, bulk formers) [14,15,16]. The management of NBD is further hindered by the presence of secondary complications such as chronic pain, requiring pharmacologic treatment. Chronic neuropathic pain, which affects approximately one-third of people with SCI, is often managed using analgesic-narcotics (opioids); unfortunately, constipation is a common side effect [17,18]. Other commonly prescribed medications that can contribute to constipation in individuals with SCI are anticholinergics [3]. 

Lynch et al. [19] suggested that in uninjured individuals, the average frequency of BM per week is estimated at 9.3, while the average for individuals with chronic SCI was estimated at 6.6. Despite the growing body of literature that focuses on various issues related to NBD following SCI [20,21], there is a paucity of data on the progression of bowel function (frequency of BM and FI) during an acute period of rehabilitation, and upon discharge to the community. It is also unknown how the use of laxatives and opioids (number of laxatives and average dose of opioids) during the acute period of rehabilitation impacts bowel management.

The objective in this study was to examine the impact of inpatient rehabilitation and the use of various medications on bowel dysfunctions of individuals with acute traumatic/non-traumatic SCI. Two questions guided our investigation: (1) How does the frequency of BM and the frequency of FI change during the acute period of rehabilitation and how does it pertain to injury-related characteristics of individuals with SCI? (2) What are the impacts of laxatives and opioids on overall bowel dysfunction during the acute period of rehabilitation of individuals with SCI?

## 2. Methods

The protocol for this study was reviewed and approved by the University of British Columbia Clinical Research Ethics Board, conforming to the Declaration of Helsinki.

### 2.1. Setting and Participants

We conducted a retrospective chart review using electronic medical records from a single, tertiary rehabilitation centre (GF Strong Rehabilitation Centre (GFSRC) in Vancouver, Canada) during a two-year period (January 2012 to December 2013) to identify a cohort of 161 patients. Only individuals with acute traumatic/non-traumatic SCI admitted for inpatient rehabilitation were included into the study. Upon admission to the rehabilitation centre, an order form with the bowel management protocol was completed for each individual. The frequency of BM, which included date and time of bowel routine was documented by nursing staff; frequency of FI was documented according to the patient report. The protocol specific to our centre included the following crucial components: diet recommendations, medications (e.g., osmotic laxatives, stimulant laxatives, suppositories and enemas) and specific bowel manipulations to assist with bowel movements (e.g., digital evacuation, digital stimulation). The decision to use diet, medication and/or various manipulations was based on the treating physician’s knowledge of UMN/LMN bowel syndrome in combination with frequency of BM and FI as documented in the patient’s chart.

### 2.2. Measures

The following demographic and injury-related characteristics were collected: age, sex, duration of SCI, mechanism of injury (traumatic vs. non-traumatic), neurological level of injury, and severity of SCI according to the International Standards for Neurological Classification of SCI (ISNCSCI) [22]. Clinical data regarding medications (including laxatives and opioids) and bowel function/management (bowel movement (BM), fecal incontinence (FI), digital stimulation (DS), digital evacuation (DE)) were measured daily during the first week of admission and the last week prior to discharge from inpatient rehabilitation. These measures were compared from admission to discharge in the whole cohort as well as in subdivision of three neurological and two functional bowel groups. The neurological level of injury groups was defined with consideration to potential motor functionality: cervical (C1-C8), thoracic (T1–T9), and lumbosacral (T10–S5). Functional bowel groups were selected based on upper motor neuron and lower motor neuron bowel syndrome: UMN (T10 and above) and LMN (T11 and below). 

Following review of the participants’ medication regimen, data on laxative and opioid use was analyzed. The dose of all opioids was converted to morphine milligram equivalents (MME) for purposes of analysis, using an established opioid analgesic conversion table [23]. None of the individuals in this study used methadone. Finally, the frequency of BM and FI were measured by calculating the mean values during the week of admission and week of discharge.

### 2.3. Statistical Analysis

Bivariate analysis of age, sex, medication use, injury severity and level was done to determine the relationship of these variables with BM status. Spearman or Pearson correlation test was used to assess association of continuous variables with outcomes and an independent t-test or Mann-Whitney U test were used to compare two groups. After the bivariate analyses, a repeated measures analysis of variance (ANOVA) was used to investigate the effect of each medication on outcome by adjusting for other demographics and clinical variables.

A *p*-value of <0.05 was considered statistically significant. All statistical analyses were performed using SPSS (version 23).

## 3. Results

### 3.1. Characteristics of Cohort of SCI Individuals

A total of 161 individuals with acute traumatic/non-traumatic SCI were included in the study. The majority of individuals in our cohort were male (112 (69.6%) and sustained traumatic cervical SCI (45.9%; Table 1). Individuals were admitted to inpatient rehabilitation on average within 52.83 ± 56.8 days of onset of SCI. The average period of inpatient rehabilitation was 79.1 ± 39.7 days.

### 3.2. Bowel Function Variables

#### 3.2.1. Changes in Bowel Function from Admission to Discharge in Whole Cohort

**Figure 2:**
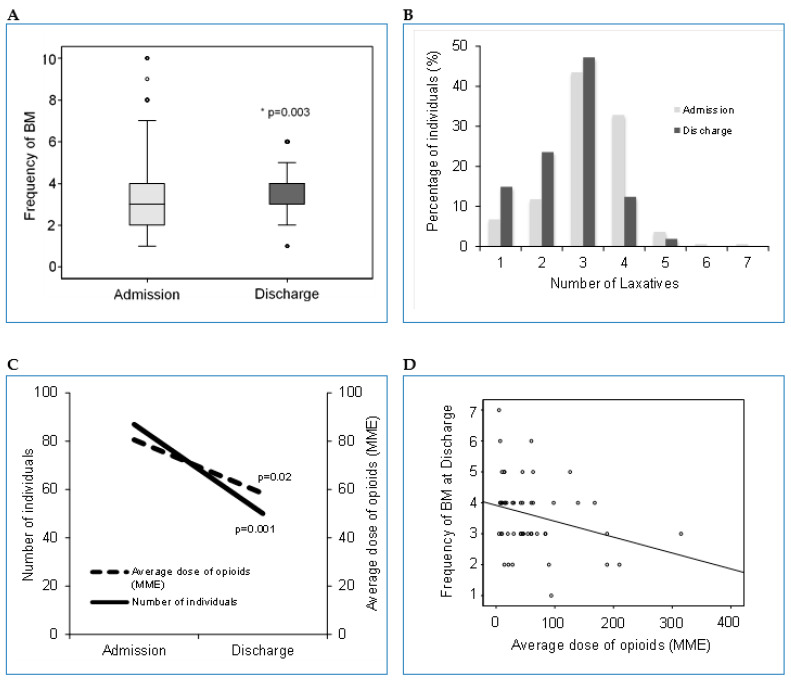
Changes in bowel function and medication use from admission to discharge in our whole cohort of individuals with SCI at an acute rehabilitation setting. (**A**) Frequency of BM at admission and discharge. (**B**) Total number of laxatives taken at admission and discharge. (**C**) Number of individuals taking opioids and average dose of opioids (MME) at admission and discharge. (**D**) Correlation between frequency of BM and average dose of opioids (MME) at discharge. Abbreviations: SCI—spinal cord injury; BM—bowel movement; MME—morphine milligram equivalents. * *p*-values < 0.01 are considered statistically significant.

When all participants were analyzed as a group, there was an increase in frequency of BM per week between admission and discharge (*p* = 0.003; Figure 2A; Table 2). There were decreases in both the number of individuals experiencing FI (78 vs. 34) as well as frequency of FI (*p* < 0.001; Table 2) between admission and discharge, respectively. There was a decrease in the number of individuals using DS between admission and discharge (71 vs. 48). Finally, there was a decrease in the number of individuals using DE between admission and discharge (70 vs. 32).

#### 3.2.2. Changes in Bowel Function from Admission to Discharge Depending on Level/Completeness of SCI

There was no difference in frequency of BM between the three neurological level of injury groups at admission or discharge (*p* = 0.46 and *p* = 0.15, respectively). There was no difference in frequency of BM within the cervical or thoracic neurological level of injury groups between admission and discharge. However, there was a significant increase in frequency of BM within the lumbosacral neurological level of injury group between admission and discharge (*p* = 0.025; Table 2).

There was no difference in frequency of FI between the three neurological level of injury groups at admission or discharge (*p* = 0.33 and *p* = 0.096, respectively). However, there was a significant decrease in frequency of FI between admission and discharge within the cervical (*p* = 0.001; Table 2), thoracic (*p* = 0.015; Table 2) and lumbosacral neurological level of injury groups (*p* < 0.001; Table 2).

There was no difference in the number of individuals using DS between the three neurological level of injury groups at admission (*p* = 0.19); however, there was a significant difference at discharge (cervical: 29, thoracic: 9, lumbosacral: 10; *p* = 0.009). There was no difference in the number of individuals using DE between the three neurological level of injury groups at admission or discharge (*p* = 0.22 and *p* = 0.175, respectively).

There was no difference between frequency of BM in motor complete vs. incomplete injury groups at admission and discharge (*p* = 0.88 and *p* = 0.06, respectively). There was no difference between frequency of FI in motor complete vs. incomplete injury groups at admission and discharge (*p* = 0.36 and *p* = 0.21, respectively). 

#### 3.2.3. Changes in Bowel Function from Admission to Discharge Depending on UMN vs. LMN Bowel Syndrome

There was no difference in frequency of BM between the two functional bowel groups at admission or discharge (*p* = 0.63 and *p* = 0.63, respectively). However, there was a significant increase in frequency of BM between admission and discharge within the UMN functional bowel group (*p* = 0.029; Table 2) and LMN functional bowel group (*p* = 0.039; Table 2).

There was no difference in frequency of FI between the two functional bowel groups at admission (*p* = 0.27); however, there was a significant decrease at discharge between the UMN and LMN functional bowel groups (0.61 ± 1.45 vs. 0.22 ± 0.62, *p* = 0.017). There was a significant decrease in frequency of FI between admission and discharge within the UMN functional bowel group (*p* < 0.001; Table 2) and LMN functional bowel group (*p* < 0.001; Table 2).

There was no difference in the number of individuals using DS between the two functional bowel groups at admission (*p* = 0.075); however, there was a significant difference at discharge (*p* = 0.002). Of those who were using DS at discharge, 85.4% were individuals in the UMN functional bowel group vs. 14.6% who were in the LMN functional bowel group. There was no difference in the number of individuals using DE between the two functional bowel groups at admission or discharge (*p* = 0.063 and *p* = 0.068); however, there does show a trend toward significance.

### 3.3. Medication Use—Laxatives

#### 3.3.1. Laxatives in Whole Cohort, Three Neurological Level of Injury Groups, and Two Functional Bowel Groups

A variety of laxatives were used between admission and discharge among the participants such as stimulant (86.3% vs. 64.6%), polyethylene glycol (42.9% vs. 31.1%) and bisacodyl suppository (32.9% vs. 29.8%). The majority of individuals in our study used laxatives during their rehabilitation at admission and discharge (150 (93%) vs. 137 (85%)). Various laxatives were commonly combined with average use of 2.19 ± 1.00 vs. 1.63 ± 0.95 at admission and discharge respectively (Figure 2B). There was a reduction in the number of individuals using laxatives (*p* = 0.004) as well as the number of unique laxatives taken (*p* < 0.0001) between admission and discharge respectively when the cohort was analyzed as a whole.

There was no difference in the numbers of individuals using laxatives between the three neurological level of injury groups at admission and discharge (*p* = 0.82 and *p* = 0.097). However, there was a significant decrease in the number of laxatives used between admission and discharge within the thoracic (*p* = 0.001; Table 3) and lumbosacral neurological level of injury groups (*p* < 0.001; Table 3).

There was no difference in the number of individuals using laxatives between the two functional bowel groups at admission. However, there was a significant difference in the number of UMN and LMN functional bowel group (82 vs. 55) laxative users at discharge (*p* = 0.016).

There was a significant decrease in the number of laxatives taken between admission and discharge within the UMN (*p* = 0.017; Table 3) and LMN functional bowel groups (*p* < 0.001; Table 3).

#### 3.3.2. Frequency of BM with Laxatives in Whole Cohort and Two Functional Bowel Groups

Regarding the whole cohort, the frequency of BM was negatively correlated with the number of laxatives used both at admission (r = −0.28, *p* < 0.001) and discharge (r = −0.16, *p* = 0.035).

Regarding the functional bowel groups, the frequency of BM was negatively correlated with the number of laxatives used at admission for the UMN (r = −0.218, *p* = 0.022) and LMN functional bowel groups (r = −0.384, *p* = 0.006). This correlation was not present at discharge in either UMN or LMN functional bowel groups.

#### 3.3.3. Frequency of FI with Laxatives in Whole Cohort

Regarding the whole cohort, there was no correlation between the number of laxatives and frequency of FI at admission; however, there was a positive correlation at discharge (r = 0.194, *p* = 0.014).

### 3.4. Medication Use—Opioids

#### 3.4.1. Opioids in Whole Cohort, Three Neurological Level of Injury Groups, and Two Functional Bowel Groups

A total of 87/161 (54.0%) and 50/161 (31.1%) from the whole cohort were taking opioid medications at admission and discharge respectively (Figure 2C). The average dose of opioids (MME) taken at admission and discharge was 80.58 ± 96.65 and 58.38 ± 63.65 respectively (Figure 2C; Table 3). The number of individuals using opioids and the average dose of opioids (MME) from admission to discharge were significantly reduced (*p* = 0.001 and *p* = 0.02, respectively).

There was a significant decrease in the number of individuals taking opioids from admission to discharge within the cervical neurological of injury group only (33 vs. 16, *p* = 0.016); however, there was no difference between admission and discharge within the thoracic (33 vs. 16, *p* = 0.20) and lumbosacral neurological level of injury groups (31 vs. 22, *p* = 0.32).

There was no difference in the average dose of opioids (MME) from admission to discharge within the cervical (*p* = 0.83; Table 3), thoracic (*p* = 0.29; Table 3), and lumbosacral levels of injury (*p* = 0.49; Table 3)

There was a significant decrease in the number of individuals taking opioids from admission to discharge within the UMN functional bowel group (61 vs. 31, *p* = 0.001); however, there was no difference for the LMN functional bowel group (26 vs. 19, *p* = 0.42).

There was no difference in the average dose of opioids (MME) taken from admission to discharge within the UMN functional bowel group; however, there was a significant decrease from admission to discharge within the LMN functional bowel group (87.50 mg vs. 60.90 mg, *p* = 0.034).

#### 3.4.2. Opioids and Frequency of Bowel Movement in the Whole Cohort and Two Functional Bowel Groups

Regarding the whole cohort, there was no correlation between average dose of opioids (MME) and frequency of BM at admission. However, there was a significant negative correlation between average dose of opioids (MME) and frequency of BM at discharge, confirming the constipating effect of opioids (r = −0.20, *p* = 0.009; Figure 2D).

In the UMN functional bowel group, there was a significant positive correlation between the frequency of BM and the average dose of opioids (MME) at admission (r = 0.350, *p* = 0.006). However, this correlation was not present in the LMN bowel group. The correlation was trending negatively in both the UMN and LMN functional bowel groups at discharge; however, it was not significant.

#### 3.4.3. Opioids and Frequency of Fecal Incontinence in the Whole Cohort and Two Functional Bowel Groups

Regarding the whole cohort, there was no correlation between average dose of opioids (MME) and frequency of FI at admission or discharge.

Regarding the functional bowel groups, there was no correlation between average dose of opioids (MME) and frequency of FI at admission or discharge for the UMN or LMN functional bowel groups.

## 4. Discussion

It is well recognized that SCI can impede the autonomic circuits responsible for normal GI function and result in the typical neurogenic bowel dysfunctions including decreased colonic motility, delayed gastric emptying, difficult defecation and FI [4,14]. To our knowledge, this is the first study to provide insight into the changes in bowel dysfunctions of individuals with acute traumatic/non-traumatic SCI during the rehabilitation period. Studies assessing bowel dysfunctions following SCI are typically conducted with chronically (>1 year) injured individuals [24]. Investigators have previously agreed that following SCI, a period of at least one year was required for bowel functions to stabilize [25,26]. Therefore, our data documents a crucial period of bowel dysfunction and subsequent bowel routine implementation during early rehabilitation and the challenge faced by these individuals and their caregivers.

Our data demonstrated that although there was an increase in frequency of BM and decrease in frequency of FI per week between admission and discharge in our cohort of individuals (Figure 2A), we were not able to detect the impact of the level and severity of SCI on these measures. Previously, Liu and colleagues [6] used the NBD score to demonstrate that the level and completeness, as well as duration of injury (>10 years), could predict the severity of NBD in individuals with chronic SCI [6]. However, other studies show conflicting data with respect to NBD [22]. A study by Pavese et al. [27] showed that the level of injury and AIS were not main predictors of severe NBD. The same investigators found that the total motor score reflected by the degree of neurological injury after SCI, was the main predictor for the severity of bowel dysfunctions following SCI, suggesting that completeness of cord injury would be a potential risk factor for severe bowel dysfunctions following SCI [27].

As evidenced by Lynch et al. [28], up to 56% of individuals with chronic SCI are affected by FI and it is crucial to recognize its negative impact on their quality of life. Our data corroborated Lynch et al. [28] in demonstrating that 48% of individuals experienced FI prior to inpatient rehabilitation, though only 21% were still experiencing FI upon discharge. Additionally, the frequency of FI in our study was significantly higher than in the study by Yim et al. [29], where investigators reported on a group of individuals with chronic SCI (injuries more than 2 years). However, our analysis of the cohort as a whole did show the frequency of FI drop dramatically from admission to discharge (1.57 ± 2.43 vs. 0.49 ± 1.26, *p* < 0.001). It is most likely that the high frequency of FI in our study was related to the early stages of rehabilitation following acute traumatic/non-traumatic SCI, as individuals are adjusting to their bowel needs and ongoing recovery of neurological functions [30]. Finally, we hypothesize that the high frequency of FI in our cohort may have been related to antibiotic-associated diarrhea that is common in SCI patients during the acute rehabilitation period [31].

As was expected, we found that there was a positive correlation between the number of laxatives and frequency of FI at discharge (r = 0.194, *p* = 0.014). This suggests that laxative use correlates with an increased frequency of FI, which was interestingly only obvious at discharge. The initiation of laxatives during acute rehabilitation after SCI is a crucial component for development of effective bowel management protocol [4]. However, clinicians need to be aware of the potential negative impact of laxatives, especially if multiple laxatives are combined with other modalities for bowel management. As evident from the study by Coggrave et al. [32], frequency of FI was significantly higher in an intervention group that used a combination of laxative and various interventions to ensure effective BM.

Similar to able-bodied individuals [33,34], opioid medications would be expected to increase CTT in individuals with SCI in a dose dependent manner and are known to result in various side effects including constipation [35]. Numerous studies examining the use of opioids following SCI have suggested chronic opioid use as less desirable [36] due to its effects on cognition [37], chronic pain [38], and tendency of habit formation. The bowel related side effects of opioids are likely related to enteric, opioid specific receptors (gamma, kappa, mu) that reduce gastrointestinal mobility through neuronal inhibition [39]. In addition to common constitutional side effects such as nausea and drowsiness observed in able-bodied individuals, opioid use in individuals with SCI can be associated with respiratory depression and further delay of gastric emptying already affected by the injury [40]. While laxatives are considered safe and are commonly included as part of bowel management protocol following SCI, their use is not innocuous. Many laxatives can result in undesirable side effects such as nausea, loose stools, abdominal cramps, excess gas, dehydration, and electrolyte imbalance [41]. Furthermore, in individuals with SCI, stimulant laxatives can be associated with unplanned bowel evacuation and an increase in the duration of time it takes to complete an evacuation [41]. As evidenced from our study, the medical team made a significant effort to decrease the number of individuals treated with opioids (54.0% at admission vs. 31.1% at discharge; Figure 2C) as well as their average dose of opioid (MME) from admission to the time of discharge. Despite the relatively large doses of opioids at admission among our cohort (average 80.58 ± 96.65 MME) we were not able to detect the impact on frequency of BM at admission. It is reasonable to expect that the risk of developing opioid induced constipation does increase over time. As demonstrated by FitzHenry et al. [42] in able-bodied individuals, the risk of opioid induced constipation is higher after six months of continuous opioid use. The majority of individuals in our cohort were admitted to acute rehabilitation within two months of injury, and therefore had only a short period of opioid exposure. However, at the time of discharge, where the majority of individuals had been using opioids for >6 months, the constipating effect of opioids was evident. This was shown by the significant negative correlation between average dose of opioids (MME) and frequency of BM at the time of discharge (r = −0.20, *p* = 0.009; Figure 2D). We postulate that additional exposures of acute illness (as in our cohort) and use of other medications could potentially increase rates of opioid-induced constipation.

We would like to acknowledge several limitations in this study. We attempted to analyze clinically relevant injuries with respect to medication use and bowel function, but due to sample size it was challenging to have sufficient power. Unfortunately, the issue of small sample size accompanies most SCI research, as this area of study pertains to relatively rare conditions of which large data are not always available. This was compounded by the incomplete data we encountered at times given the study’s retrospective nature. Finally, lumbosacral injuries were particularly underrepresented in our patient sample, which limited us in intergroups comparison. However, the low number of patients with lumbosacral level of injury reflects the epidemiology of SCI, where only 11–22% of individuals present with lumbosacral injuries [43,44,45].

The authors would also like to acknowledge that the selected LMN functional bowel group in this study (T11 and below) may potentially include a combination of mixed and LMN injury. While the group of individuals with T10 and above had true UMN bowel dysfunction.

The authors also recognize the fact that multiple factors could influence bowel function including activity level, fluid intake, and diet. We did not collect information regarding food and oral liquid intake in our study, factors known to contribute to constipation. However, it has to be noted that all individuals in this study were on a hospital-based diet and inpatient bladder management protocol in which patients were given at least 2000 cc of fluid per day. With respect to activity level, all individuals during inpatient rehabilitation for acute traumatic/non-traumatic SCI have very similarly structured rehabilitation activities depending on their level of injury. Furthermore, their physical activity outside of structured therapy within the inpatient SCI rehabilitation setting is relatively low as a substantial amount of time is spent in sedentary leisure time activities [46].

Acknowledging the great distress that bowel dysfunction contributes to the quality of life of those with spinal cord injury, it is unfortunate we could not collect data on the satisfaction of medication use, bowel dysfunction, and implemented bowel retraining.

## 5. Conclusions

In conclusion, we examined bowel function evolution during the sensitive time of acute rehabilitation. NBD presents significant challenges for individuals with SCI and medical professionals in the community. Clinicians should be aware of the negative impact of laxatives, especially if multiple laxatives are being combined with other modalities for bowel management. Opioid use should be minimized where possible; however, many individuals still require opioid analgesics for pain management. As this study was restricted to the acute traumatic/non-traumatic SCI patients, further research studying laxative use, opioid use, and bowel dysfunction in individuals with chronic SCI is needed.

## Figures and Tables

**Table 1:** Demographic characteristics of participants.

Characteristics	Mean ± s.d. or N (%)
Total number of participants Age (Years)	161 (100.0%) 48.1 ± 19.1
Sex (Male/Female)	112 (69.6%)/49 (30.4%)
Time since injury (Days)	52.8 ± 56.8
Mechanism of injury (% Traumatic)	103 (64.0%)
Neurological level of injury on admission	
Cervical	74 (45.9%)
AIS A + B	25 (15.5%)
AIS C + D	49 (30.4%)
Thoracic	39 (24.2%)
AIS A + B	21 (13.0%)
AIS C + D	18 (11.2%)
Lumbosacral	48 (29.8%)
AIS A + B	18 (11.2%)
AIS C + D	30 (18.6%)
Neurological level of injury at discharge	
Cervical	69 (42.8%)
AIS A + B	20 (12.4%)
AIS C + D	49 (30.4%)
Thoracic	34 (21.1%)
AIS A + B	13 (8.1%)
AIS C + D	21 (13.0%)
Lumbosacral	58 (36.1%)
AIS A + B	12 (7.5%)
AIS C + D	45 (28.0%)
AIS E	1 (0.6%)
Functional bowel levels on admission	
UMN	119 (73.9%)
LMN	42 (26.1%)
Functional bowel levels at discharge	
UMN	111 (68.9%)
LMN	50 (31.1%)

Abbreviations: s.d—standard deviation; N—number of participants; AIS—ASIA Impairment Scale.

**Table 2:** Change in bowel dysfunctions from admission to discharge in individuals with SCI during acute rehabilitation.

Frequency	Whole Cohort	Neurological Level of Injury Groups			Functional Bowel Groups	
		Cervical	Thoracic	Lumbosacral	UMN	LMN
	Admission vs. Discharge, *p*-Value					
BM (Mean ± s.d)	3.38 ± 1.85 vs. 3.84 ± 1.15, *p* = 0.003	3.39 ± 1.84 vs. 3.83 ± 1.16, *p* = 0.113	3.59 ± 2.10 vs. 4.06 ± 1.59, *p* = 0.174	3.24 ± 1.73 vs. 3.74 ± 1.07, *p* = 0.025	3.43 ± 1.88 vs. 3.87 ± 1.18, *p* = 0.029	3.26 ± 1.73 vs. 3.78 ± 1.11, *p* = 0.039
FI (Mean ± s.d)	1.57 ± 2.43 vs. 0.49 ± 1.26, *p* < 0.001	1.72 ± 2.59 vs. 0.71 ± 1.59, *p* = 0.001	1.62 ± 2.85 vs. 0.5 ± 1.26, *p* = 0.015	1.34 ± 1.93 vs. 0.22 ± 0.62, *p* < 0.001	1.69 ± 2.56 vs. 0.61 ± 1.45, *p* < 0.001	1.21 ± 1.92 vs. 0.22 ± 0.62, *p* < 0.001

Abbreviations: BM—bowel movement; FI—fecal incontinence; UMN—upper motor neuron; LMN—lower motor neuron; s.d.—standard deviation.

**Table 3:** Change in laxative and opioids use from admission to discharge in individuals with SCI during acute rehabilitation.

Medication	Whole Cohort	Neurological Level of Injury Groups			Functional Bowel Groups	
		Cervical	Thoracic	Lumbosacral	UMN	LMN
	Admission vs. Discharge, *p*-Value					
Laxatives (Mean ± s.d)	2.19 ± 1.00 vs. 1.63 ± 0.95, *p* < 0.001	2.00 ± 0.92 vs. 1.83 ± 0.89, *p* = 0.159	2.35 ± 1.04 vs. 1.68 ± 0.98, *p* = 0.001	2.33 ± 1.03 vs. 1.36 ± 0.95, *p* < 0.001	2.09 ± 0.98 vs. 1.81 ± 0.90, *p* = 0.017	2.32 ± 1.01 vs. 1.39 ± 0.96, *p* < 0.001
Opioids (Average MME ± s.d)	80.58 ± 96.65 vs. 58.38 ± 63.65, *p* = 0.02	58.30 ± 56.61 vs. 43.74 ± 46.58, *p* = 0.83	109.37 ± 143.12 vs. 61.58 ± 56.44, *p* = 0.29	82.94 ± 84.57 vs. 67.28 ± 77.35, *p* = 0.49	99.10 ± 110.41 vs. 70.97 ± 72.61, *p* = 0.13	87.50 ± 73.64 vs. 60.90 ± 57.88, *p* = 0.034

Abbreviations: UMN—upper motor neuron; LMN—lower motor neuron; s.d.—standard deviation; MME—morphine milligram equivalents.

## Data Availability

The data presented in this study are available on request from the corresponding author. The data are not publicly available due to their containing information that could compromise the privacy of research participants.
